# The landscape of dementia in India: Prevalence, risk factors, and opportunities ahead

**DOI:** 10.1002/alz.71526

**Published:** 2026-06-01

**Authors:** Akankshi Oberoi, Karen Cai, Rebecca West, Anju Wadhawan, Auriel Willette, Sharon Sanz Simon, Jack Tsao, Yian Gu, Luciana Mascarenhas Fonseca, Tushar Patel, Michal Schnaider Beeri

**Affiliations:** ^1^ Rutgers Krieger Klein Alzheimer's Research Center New Brunswick New Jersey USA; ^2^ Rutgers School of Nursing Newark New Jersey USA; ^3^ Columbia University Irving Medical Center New York New York USA

**Keywords:** Alzheimer's disease, dementia, health disparities, India, risk factors

## Abstract

India's rapidly aging population faces a dementia burden, yet data on factors affecting dementia risk remain limited. A narrative review of studies on dementia prevalence and risk factors in India was conducted using Medline, Scopus, and Google Scholar. Findings were synthesized to examine the 14 modifiable risk factors identified by the 2024 Lancet Commission. The Indian dementia research landscape remains dominated by cross‐sectional studies. The prevalence of dementia is lower in India than in Western cohorts. However, high prevalence is observed among women, rural dwellers, and individuals with cardiovascular comorbidities, especially diabetes, a condition exhibiting unique metabolic phenotypes within this population. Undocumented alcohol use, air pollution, traumatic brain injuries, loneliness, depression, and sensory deficits are prevalent across India, yet literature linking these exposures to dementia risk lacks empirical research. There is an urgent need for longitudinal studies to identify modifiable risk and protective factors for dementia in the Indian population.

## Highlights

- India's rapidly aging population faces a growing dementia burden
- Metabolic and vascular risk factors play a key role in midlife dementia risk
- Social, cultural, and lifestyle factors shape dementia presentation
- Lack of India‐specific literature limits understanding of dementia risk factors
- Early prevention targeting modifiable risks is key to reducing dementia burden

## 1 INTRODUCTION

An aging population is a global phenomenon and has become a significant public health challenge in many low‐ and middle‐income countries (LMICs) in Asia.^1^ India, the world's most populous country, is undergoing a rapid demographic transition marked by a steady rise in its older adult population.^2^ The United Nations Population Fund forecasts that the elderly population in India will reach 340 million by 2050, accounting for 17% of the global population 60 years of age and older.^3^ The World Health Organization and the United Nations have designated 2021–2030 as the “Decade of Healthy Aging,” emphasizing global efforts to promote health and well‐being in later life.^4^

Dementia is a progressive condition characterized by impairments in cognitive abilities, including learning, memory, attention, language, and decision‐making, as well as changes in behavior, personality, and mood. The clinical spectrum of cognitive decline ranges from mild cognitive impairment (MCI) to severe dementia that progresses and leaves the patient unable to carry out daily tasks without assistance, ultimately leading to death.^5^ Advancing age is the most substantial risk factor for dementia, but dementia is not an inevitable part of aging.^6^ The 2024 Lancet Standing Commission reported 14 modifiable risk factors for dementia—lower education, hearing loss, hypertension, smoking, obesity, diabetes, excessive alcohol use, traumatic brain injury (TBI), physical inactivity, high low‐density lipoprotein cholesterol (LDL‐C), social isolation, air pollution, untreated vision loss, and depression—based on global prevalence estimates. Addressing these factors could potentially prevent or delay up to 45% of dementia cases worldwide.^7^ However, most evidence comes from studies conducted in high‐income countries (HICs), including the United States, Canada, the UK, and Europe. Differences in genetics, diet, sociodemographics, health care practices, and health education make it uncertain how directly these estimates apply to LMICs, such as India.^8^

Despite the expected surge in dementia cases in India, a limited body of literature has summarized the evidence on these risk factors in the Indian context.^9^ To address this gap, this review examines the literature on the prevalence and risk factors of dementia in India. We summarize evidence from India on the 14 modifiable risk factors identified by the 2024 Lancet Commission.^7^

## 2 METHODS

A review of the literature published between 2000 and 2025 was conducted to synthesize the evidence on the prevalence of dementia and associated risk factors in India. Relevant studies were identified through searches in MEDLINE, Scopus, and Google Scholar using combinations of the keywords “dementia,” “Alzheimer's disease,” “India,” and “epidemiology.” Additional articles were located through the reference lists of pertinent reviews. Studies focusing on Indian populations and reporting prevalence estimates or modifiable risk factors were included. Findings were organized thematically according to demographic and contextual patterns observed across studies in Table 1.^10^, ^11^, ^12^, ^13^, ^14^, ^15^, ^16^, ^17^, ^18^, ^19^, ^20^ The 2024 Lancet Commission framework, which identifies 14 modifiable risk factors for dementia, served as the baseline for categorizing and interpreting findings from the Indian context.

**TABLE 1:** Characteristics and key findings of epidemiological studies on dementia in India.

Study	Year of analysis	Region	Age group	Sample size	Overall dementia prevalence	Prevalence by sex	Prevalence by urbanicity	Risk factors
Lee et al.^10^	2018–2020	National	>60	31477	7.43%	M, 5.77%	Urban, 5.34%	Older age
						F, 9.03%	Rural, 8.35%	Female sex
								No formal education
Tiwari et al.^11^	2008–2010	Uttar Pradesh	≥60	2146	2.8%	M, 0.82%	Rural	Older age
						F, 1.95%		Female sex
								Lower socio‐economic status
Poddar et al.^12^	2011	Eastern Uttar Pradesh	>60	2890	5.1%	M, 3.8%	Urban, 3.8%	Older age
						F, 7.2%	Rural, 5.5%	Female sex
								Lower education
								Family status, not married
								Unemployment
								Family size <16
Gambhir et al.^13^	2007	Uttar Pradesh	>60	728	2.74%	M, 2.7%	Rural study	Older age
						F, 2.8%		Illiteracy
								Female sex
								Undernutrition
Gurukartick et al.^14^	2013–2014	Tamil Nadu	≥65	1300	3.1%	M, 3.8%	Rural study	Older age
						F, 2.4%		Male sex
								At least 1 cardiovascular risk factor
								Above poverty line
								No previous involvement in family decisions
Shaji et al.^15^	2018	Kerala	≥65	1934	3.36%	M, 3.43%	Urban study	Older age
						F, 2.45%		Family history
								Hypertension
Mathuranath et al.^16^	2004	Kerala	≥55	2466	Age 55+	Age 55+	Urban study	Older age
					3.77%	M, 2.79%		
					Age 65+	F, 4.44%		Female sex
						Age 65+		Lower education (≤8 years)
					4.86%	M, 3.25%		
						F, 5.02%		
Raina et al.^17^	2008	Kashmiri migrants	≥60	200	6.5%	M, 8.4%	N/A	Older age
						F, 4.7%		Male sex
C J Vas et al.^18^	2001	Mumbai	≥40	24488	0.41%	M:F, 0.75:1 and 1:0.69 for AD and VaD, respectively.	Urban study	Older age
Raina et al.^19^	2014	Himachal Pradesh	≥60	2000	1.6% overall	M, 0.55%	Urban > Migrants > Rural > Tribal	Older age
					0% in tribal elderly	F, 1.05%		Urbanicity
Raina et al.^20^	2010	Jammu & Kashmir	≥60	1856	1.83%	M, 1.6%	Rural study	Older age
						F, 1.99%		Female sex
								Migrants

Abbreviations: AD, Alzheimer's Dementia; F, Females; M, Males; N/A, Not Available; VaD, Vascular Dementia.

## 3 DISCUSSION

### 3.1 Demographic differences in the prevalence of dementia in India

#### 3.1.1 Sex

Women in India, similar to those in Western countries, face a higher risk of dementia.^10^, ^11^, ^12^, ^16^, ^20^ In LMICs, certain exposures disproportionately affect brain health in women, whereas others are more detrimental for men. For women, factors such as limited education, female genital mutilation, child marriage, early‐life physical and sexual violence, low political empowerment, and occupational inequality contribute significantly to risk. In contrast, men are more adversely affected by exposures including occupational TBI, war and conflict, and midlife cardiometabolic risk factors.^21^ Understanding gender‐specific factors in Indian society is crucial for addressing this disparity and reducing the risk of dementia. Multiple interrelated factors, including lower literacy rates, undernutrition, and entrenched social discrimination, may contribute to the heightened vulnerability among women.^22^ In India, the sex gap in literacy is significant, with only 62.3% of women being literate, compared to 80% of men.^23^ This educational disadvantage, rooted in cultural norms, limits women's access to health information and care, potentially influencing cognitive health outcomes in later life. Cultural perceptions surrounding girls’ education further reinforce this gender disparity. In many Indian families, daughters are viewed as financial liabilities due to dowry and marriage costs, while sons are valued for their religious and social roles.^23^, ^24^ Consequently, parents, especially in lower‐ to middle‐income households, often deprioritize investment in daughters’ education, believing that any benefits will accrue to their future marital families rather than their families of origin. These persistent gender biases in social and familial structures may play a significant role in shaping women's long‐term health and cognitive outcomes.^24^

Of interest, two studies in Table 1, one in rural Tamil Nadu and among Kashmiri migrants, found a higher prevalence of dementia in men.^14^, ^17^ Although men 65 years or older in rural Tamil Nadu tend to be more educated and play a greater role in family decision‐making, dementia prevalence remains high in this group. This paradox may reflect reduced engagement in structured, socially, or cognitively stimulating activities after stopping occupational work. Among Kashmiri migrants, displacement resulted in the loss of occupational roles, community ties, and social structure, particularly affecting older men. Women's continued engagement in daily household activities may have provided cognitive stimulation that is protective. These findings highlight that the influence of social context and life transitions can shape gender differences in dementia risk.^22^

#### 3.1.2 Urbanicity

Similar to the Western world,^25^ rural areas have shown a higher prevalence of dementia in studies conducted in India,^10^, ^12^ except for one study which reported a contrasting trend.^19^ A meta‐analysis of Indian studies reported a 50% higher prevalence of dementia in rural as compared to urban older adults.^26^ This finding is alarming because, currently, 65% of the Indian population resides in rural areas. However, the degree of urbanization has increased in recent years.^27^ Disentangling what contributes to a higher risk of dementia in rural areas is complex. For example, two cohort studies in India reported a higher prevalence of hypertension (59%), diabetes mellitus (34%), and obesity (81%) in the urban population, whereas the rural population exhibited a lower prevalence of these conditions (31%, 17%, and 46%, respectively).^28^ However, the prevalence of undiagnosed cases is significantly higher in rural populations than in urban populations: 62.6% versus 32.1% for hypertension and 26.7% versus 10.9% for diabetes.^29^ This higher rate of undiagnosed diseases in rural areas was attributed to lower levels of education, lower socioeconomic status, low body mass index (BMI), and limited health awareness. Although urban areas may have a greater burden of diagnosed chronic diseases that increase the risk of dementia, the greater proportion of undetected conditions in rural settings highlights the critical need for improved awareness, screening, and health care access to reduce dementia risk in these populations.^28^

### 3.2 Prevalence of modifiable risk factors identified by the 2024 Lancet Commission

#### 3.2.1 Education

Consistent with findings from Western countries, in India, low education has been associated with a higher prevalence of dementia.^10^, ^12^, ^13^, ^16^, ^30^ It accounts for the largest proportion of population attributable fraction (PAF) for dementia in many LMICs, in contrast to HICs.^31^ Therefore, education has a greater potential to decrease the burden of dementia in LMICs, including India. The definition of “low education” as a risk factor for dementia varies significantly depending on the population being studied. In HICs, where most adults have completed secondary education and illiteracy is rare, “low education” is typically defined as not completing high school or having fewer than 12 years of schooling.^32^ In contrast, many LMICs have large populations with limited access to education. To better reflect the Indian context, some researchers have classified “no formal education” as the risk factor for dementia, rather than using the standard cutoff of less than secondary education that is common in high‐income settings.^33^ This tailored approach to a particular environment is critical, as education levels are a significant risk factor for cognitive decline and incident dementia.

Globally, low education accounts for 8% of potentially modifiable dementia risk factors, but in India, it accounts for 22%.^33^ This higher contribution may be driven by greater educational disparities, limited health care awareness, and reduced cognitive stimulation among those with lower levels of education. This reflects the robust role education plays in building cognitive reserve in these settings. A study in India reports that dementia prevalence rates among illiterates versus those with elementary school (up to fifth grade) were 12.6% and 3.4%, respectively.^13^ The preventive impact of education in India might reshape the Lancet Commission model into a notably stronger influence in dementia prevention.^33^

#### 3.2.2 Type 2 Diabetes

South Asia is a primary region affected by the type 2 diabetes epidemic.^34^ India ranks among the top three countries worldwide, alongside China and Pakistan, in both the absolute numbers of adults affected with diabetes and the number of undiagnosed cases.^35^ In 2021, India was estimated to have over 74.2 million adults living with diabetes, and this number is projected to reach nearly 125 million by 2045.^35^ These figures highlight a national crisis but also offer essential insights into broader health trends across the South Asian region, as India accounts for the majority of the region's population.^36^ The prevalence of diabetes in India is 16% as compared to 13% in the United States.^37^, ^38^ More importantly, some evidence shows that having diabetes confers a higher risk of dementia in South Asians compared to Whites.^39^ The South Asian phenotype of diabetes has a distinct pattern of clinical features, which could contribute to this difference in effect on dementia risk. It is characterized by higher insulin resistance, central obesity, and increased visceral fat, despite a normal or low BMI, a pattern often referred to as the “thin‐fat” Indian phenotype. These individuals tend to develop type 2 diabetes at a younger age and lower body weight compared to the Western populations, in whom diabetes is more directly associated with obesity.^40^, ^41^ In addition, Indians progress more rapidly from prediabetes to diabetes. Therefore, early screening and culturally tailored interventions are crucial for effective management of diabetes and, therefore, dementia.^40^

Midlife onset of diabetes is consistently associated with an increased risk of developing dementia in HICs.^42^ Despite India's status as a global epicenter of diabetes, few studies have examined the association between diabetes and dementia risk in the Indian context. The cross‐sectional analysis from LASI‐DAD and HRS‐HCAP demonstrated that elevated glycated hemoglobin (HbA1c) was associated with a higher odds of significant cognitive decline among Indians. This could likely reflect socioeconomic confounding and differential access to health care.^43^, ^44^ This gap is noteworthy given that individuals with diabetes in India may be at a disproportionately higher risk of dementia compared to those in HICs. Factors such as earlier age at diabetes onset, poorer glycemic control, a higher prevalence of comorbid vascular risk factors (e.g., hypertension and dyslipidemia), and sociocultural determinants (including lower health literacy and potential differences in health care access and management), may amplify this risk. Despite these concerns, the lack of large‐scale longitudinal studies in the Indian population limits the ability to establish a clear causal relationship between diabetes and dementia, underscoring the need for prospective research in this setting.^39^

#### 3.2.3 High LDL

A cross‐continental study found that mean LDL‐C levels were lower in India (102.8 mg/dL) as compared to Europe (123.7 mg/dL) and the United States (108.3 mg/dL).^45^ Compared to the White population, Asian Indians are disproportionately characterized by a more atherogenic lipoprotein profile, with a higher prevalence of small dense LDL particles, which are more atherogenic and more strongly associated with cardiovascular risk than larger LDL particles.^46^, ^47^ It highlights the importance of recognizing the distinct and potentially higher‐risk lipid phenotype in Asian Indians compared to other communities worldwide.

Multiple extensive studies have found that higher LDL‐C is associated with an increased risk of dementia.^9^ A systematic review and meta‐analysis of studies from HICs, including adults <65 years, reported an 8% rise in all‐cause dementia risk for every 1 mmol/L increase in LDL‐C.^48^ Conversely, lower LDL‐C levels (<70 mg/dL) are associated with reduced Alzheimer's risk, and statin use has been linked to a lower risk of developing dementia.^49^ However, clinical trials of statins have not demonstrated clear cognitive benefits, and the relationship between high LDL‐C and dementia risk in late life remains uncertain. There is a lack of research specifically examining whether LDL levels are related to dementia risk in the Indian population.

#### 3.2.4 Hypertension

The National Survey from India reports an overall hypertension prevalence of 28% as compared to 47.7% in the United States, as per the Centers for Disease Control and Prevention (CDC).^50^, ^51^ However, the diagnostic cutoff for hypertension is lower in the United States. Considering this, the prevalence of hypertension in India, as per the latestThe American College of Cardiology (ACC) and American College of Cardiology (AHA) guidelines (≥130/80 mmHg), was much higher (63.8%), making hypertension a significant public health concern in India.^52^

Studies from HICs have consistently shown that hypertension is linked to an elevated risk of late‐life cognitive impairment and dementia.^53^ A clinical trial involving hypertensive individuals aged 50 years and older in the United States demonstrated that, with a mean follow‐up of 5.1 years, the intensive blood pressure management group saw a 17% reduction in the primary endpoint (probable dementia).^54^

The association between hypertension and dementia remains underexplored in India. We have found a few studies suggesting an association between higher blood pressure and dementia in India.^14^, ^15^ Given India's rapidly aging population and rising burden of non‐communicable diseases, there is a pressing need to understand the link between hypertension and dementia in India, which could inform early screening and intervention strategies aimed at dementia prevention.^55^

#### 3.2.5 Obesity

Studies from HICs suggest that more obesity, typically measured by BMI, is associated with a higher dementia risk. Similarly, abdominal obesity, measured by waist circumference, has also been linked to higher dementia risk, with stronger effects among women than men.^56^, ^57^ Obesity accounts for higher PAF in HICs as compared to LMICs.^31^ The relationship in India appears more complex and context dependent, with both undernutrition and differences in body composition influencing dementia risk across the lifespan.^33^ In comparison to White populations, South Asian populations often have higher body fat percentages at lower BMIs. Indians may already be at risk for poor health outcomes (such as diabetes or hypertension) due to BMIs considered “normal” by Western standards.^58^ Therefore, the Association of Physicians in India has recommended an alternative, more conservative classification for the Indian context: BMI <18 kg/m^2^ for underweight, 18 ≤ BMI <23 for normal, 23 ≤BMI < 25 for overweight, and BMI ≥25 for obese, as compared to HICs, which define underweight as <18.5 and obese as ≥30.^30^ According to these obesity definitions, the prevalence of obesity in the United States was reported to be 42.4% of the overall adult population, whereas in India, it is ≈43%.^59^, ^60^

The LASI study found that obesity was associated with lower dementia risk when defined by the standard BMI threshold (≥30), but not when using the Indian‐specific BMI threshold (≥25). It also showed an association of underweight (BMI <18) with higher dementia risk. These unexpected findings may reflect survival bias or reverse causation (where weight loss precedes dementia), which could confound cross‐sectional studies or reflect unique population characteristics in India.^33^ Notably, this association lost statistical significance among urban populations and individuals with higher educational attainment, suggesting that factors such as urbanicity and education may influence the relationship between obesity and dementia risk in India. These findings highlight potential contextual differences between HICs and India, where malnutrition remains more common, pointing to a complex, non‐linear relationship between BMI and dementia. However, more studies are needed to better understand these associations in the Indian setting.

#### 3.2.6 Physical inactivity and exercise

The Lancet Global Health study on physical inactivity reveals that the global rate of insufficient physical activity rose from 26.4% in 2010 to 31.3% in 2022. The most pronounced increase was observed in South Asia (32.1% in 2010 to 45.4% in 2022), whereas Western nations showed a decline (30.1% to 27.7%) in the prevalence of physical inactivity over the same period.^61^ The EXERT study (Exercise in Adults with Mild Memory Problems), a multisite clinical trial, suggested that both lower‐ and higher‐intensity exercise could slow cognitive decline.^62^ Even just 35 min of moderate to vigorous physical activity per week has been shown to lower the risk of developing dementia by 41% over an average follow‐up period of 4 years.^63^

LMICs have a higher PAF for inactivity than HICs, and therefore, reducing sedentary behavior may have a greater potential for preventing dementia.^31^ In India, physical inactivity among adults remains a significant concern, with 57% of adults failing to meet the World Health Organization's (WHO) recommended levels of physical activity, a rate considerably higher than those reported in the United States (25.3%) and Europe (36.2%).^64^, ^65^, ^66^ Urban adults in India are found to be less active than their rural counterparts.^64^, ^67^ The regional disparity may be attributed to higher income, sedentary work environments, and greater access to mechanized transport and modern household appliances among urban populations. The majority of physical activity among Indian adults, especially in rural areas, is work related, whereas leisure‐time physical activity is almost non‐existent for over 90% of the population.^67^ Despite over half of Indian adults being physically inactive, there is a lack of longitudinal studies investigating the impact of physical inactivity on dementia risk in India. This trend has significant public health implications, particularly since work‐related activity often declines after retirement. Addressing physical inactivity could potentially prevent a substantial proportion of dementia cases.^68^ Strategies to promote physical activity among older adults could include community‐based exercise programs, public awareness campaigns, and improving access to safe recreational spaces. Given the growing population of older adults in India, implementing such interventions is crucial for mitigating the future burden of dementia.

#### 3.2.7 Social isolation

Loneliness is a subjective emotional experience that arises when there is a gap between a person's desired and actual quality or quantity of social relationships. In contrast, social isolation is an objective condition characterized by limited social contact, typically measured by the size of an individual's social network and the frequency of their interactions with others.^69^ The initial phase of the LASI in 2017–2018 revealed that 20.5% of adults 45 years of age and older experienced moderate loneliness.^70^ Overcrowding in India can heighten emotional distress by limiting personal space and encouraging disconnection. In contrast, the strong emphasis on extended family systems, cultural gatherings, and community support helps reduce social isolation.^71^ Festivals and religious events provide opportunities for social engagement, reinforcing a sense of belonging.^12^, ^14^ As urbanization increases, understanding how population density affects social isolation becomes increasingly essential.

Low social participation, less frequent social contact, and more loneliness are consistently associated with incident dementia in HICs.^72^] A one‐unit increase in participation in a social activity was associated with a 13% decrease in the odds of MCI in LMICs, including India.^73^ Involvement in family decisions and a larger family size were associated with better cognitive functioning in India.^12^, ^14^ HIC has implemented various community‐based interventions, such as senior centers and telehealth programs, to combat isolation, integrating these strategies into broader dementia prevention efforts.^74^ However, social isolation remains a largely underrecognized issue in dementia research and care in India, with limited structured interventions aimed at mitigating its effects.^75^ Although research on loneliness and social participation with dementia risk in India has been minimal, the direction of the associations is similar to those found in HICs. This reflects the need for more studies investigating its role in the Indian community, to provide the necessary evidence for the development of more comprehensive, culturally appropriate interventions and increase awareness of social isolation as a modifiable dementia risk factor.

#### 3.2.8 Depression

In the United States, the lifetime and current prevalence of depression among adults (>18 years) are estimated at 29% and 17.8%, respectively.^76^ In contrast, the latest National Mental Health Survey (2015–2016) reported significantly lower rates in India, with lifetime and current prevalence estimated at 5.25% and 2.68%, respectively. These figures likely underestimate the actual burden, as depression in India is frequently underdiagnosed and undertreated due to factors such as cultural stigma, limited awareness, and inadequate access to mental health care.^77^ In the Indian context, where depression is often underdiagnosed in older adults, these insights reinforce the importance of integrating mental health screening into routine geriatric care. One significant barrier to effective depression care in India is the persistent stigma surrounding mental health, which often leads to misunderstanding, fear, and reluctance to seek help.^78^

The relationship between depression and dementia is complex and appears to be bidirectional. Depression may act as a risk factor preceding the onset of dementia, but it can also emerge as an early symptom or psychological response to developing cognitive impairment. Depression can further lead to social isolation, which is in itself a risk factor for dementia.^76^ Longitudinal studies from HICs have demonstrated that depression at any stage of life is associated with an elevated risk of developing dementia.^79^, ^80^ Depression is significantly associated with dementia in India, although the strength and nature of this relationship have not been studied as extensively as in HICs.^81^ The specific patterns of onset, severity, and progression of depression, along with how these factors are linked to dementia risk, remain poorly understood in the Indian context. Future research investigating these associations could support policy changes to prioritize mental health, promote early identification and treatment of depression, and address its important role in cognitive decline and dementia.

#### 3.2.9 Smoking

Smoking is associated with an increased risk of dementia.^7^ According to the 2022 CDC report, 11.6% of U.S. adults are current cigarette smokers.^82^ In India, the smoking prevalence rate among individuals aged 15 or older is 7.1%. The rate varies widely among men (12.8%) and women (1.1%).^83^

The cohort study in London found a higher dementia risk among current smokers as compared to ex‐smokers or non‐smokers.^84^ A meta‐analysis reported that current smokers have a 1.30 times higher risk of all‐cause dementia and 1.40 times higher risk of Alzheimer's disease.^85^ Evidence linking smoking to dementia in India is mixed. A cross‐sectional study across LMICs, including India, found a significant association between smoking and higher dementia prevalence.^86^ However, a 10/66 longitudinal cohort analysis found no significant associations.^87^ Similarly, another cross‐sectional study using the Harmonized Diagnostic Assessment of Dementia for the LASI (LASI‐DAD), in contrast with findings from HICs, did not show statistically significant associations between smoking and dementia.^33^ There is currently a lack of literature examining the relationship between smoking and dementia risk in Indian populations. However, LMICs bear the greatest PAF for smoking, which declines with increasing income levels.^31^ This emphasizes the need for long‐term research to disentangle the link between smoking behaviors and dementia in the Indian context.

#### 3.2.10 Alcohol

In India, the average alcohol consumption per year in 2022 was 4.5 L, as compared to 9.8 L in the United States.^88^ According to National Family Health Survey‐5 (NFHS‐5; 2019–2021), alcohol consumption in India is significantly higher among men (18.8%) than women (1.3%) ages 15–49. It is more prevalent in rural areas and among Scheduled Tribes (officially recognized indigenous communities in India), with increased rates observed in men with lower education levels, particularly those 35–49 years of age.^89^ Of interest, 51% of all alcohol consumed in India is unrecorded, especially among rural and disadvantaged groups, which is home‐brewed or illicitly made, often in unsafe conditions without quality control. These unregulated drinks, known as desi daru or country liquor, may contain neurotoxins like methanol, lead, or arsenic due to poor distillation and storage. This unrecorded alcohol production may be related to a higher dementia risk compared to regulated commercial alcohol.^90^

Evidence on the relationship between alcohol and dementia risk is mixed. Compared with abstention, the consumption of 1 to 6 drinks weekly is associated with a lower risk of incident dementia, whereas >6 drinks is associated with a higher risk in a case‐control study conducted in the United States.^91^ A cohort study in the United States found that light (1–2 drinks/day) daily drinking lowered dementia risk in individuals without MCI, but heavy drinking (>14 drinks/week) worsened decline in those with MCI.^92^There are some studies from India that reported associations between alcohol consumption and a higher risk of cognitive impairment.^93^, ^94^ However, the relationship between drinking patterns and dementia risk in India has not been investigated yet. Future studies are needed to better understand how alcohol impacts cognitive decline and dementia risk within the Indian context.

#### 3.2.11 Air pollution

The WHO considers particulate matter (PM) 2.5 levels above 5 µg/m^3^ to be excessive, whereas India's air quality standards permit an annual average of up to 40 µg/m^3^.^95^ It is alarming that pollution levels in India have an overall annual geographic mean of PM 2.5, rising from 27 µg/m^3^ in 1998 to 44 µg/m^3^ in 2022.^96^ Although India is among the most polluted countries in the world, there is a notable lack of longitudinal research specifically linking air pollution to dementia incidence in India. Epidemiological studies show that air pollution, particularly fine particulate matter (PM2.5 and PM10) and nitrogen dioxide (NO_2_), is consistently associated with increased risk of dementia, including Alzheimer's disease, across diverse populations and countries.^97^, ^98^ Collectively, the evidence highlights air quality improvement as a crucial and modifiable public health strategy to reduce the risk of dementia.

In the Indian context, indoor air pollution remains a significant environmental exposure that warrants consideration in dementia risk assessments.^99^ Despite the Ministry of Petroleum and Natural Gas reporting a 99.8% liquefied petroleum gas coverage, the NFHS‐5 (2019–2021) indicates that 41% of households still use biomass such as wood, cow dung, and agricultural residue for cooking purposes.^96^ Biomass fuel emits PM and nitrogen oxides, which have been defined by the Lancet Commission as risk factors for dementia.^8^ Because women are primarily responsible for cooking and older adults spend most of their time indoors, they are the individuals most exposed to these risk factors.^100^ Given the high levels of both indoor and outdoor air pollution in India, there is a pressing need for comprehensive longitudinal studies that investigate in depth their associations with dementia risk.

#### 3.2.12 Vision impairment

Although the 2024 Lancet Commission does not specify precise visual acuity cutoffs, it relies on large‐scale cohort evidence showing that persisting, uncorrected vision impairment, ranging from presbyopia (a form of refractive error) to moderate‐to‐severe acuity deficits and blindness, is associated with an increased risk of dementia.^8^ Crude prevalence of all‐cause vision impairment (including blindness, distance, and near vision impairment) in adults aged 50 years or older is significantly higher in India (75%) as compared to the United States (11%) and the UK (13%).^101^ Vision impairment accounts for 14% of prevalent dementia cases in India, making it one of the most significant modifiable risk factors.^33^ This contrasts with HICs, which have the lowest amount of PAF due to visual loss.^31^ Government‐led initiatives, including subsidized eye check‐ups, screening for diabetics, the use of corrective lenses, and timely cataract surgeries, reflect India's commitment to reducing preventable vision loss and ensuring that quality eye care is accessible to all citizens.^102^ However, there is significant underutilization of these services, likely due to several barriers, including limited access to eye care services in rural and underserved areas, such as low public awareness, a shortage of trained personnel, and high out‐of‐pocket costs for corrective interventions.^103^

A cohort from the UK has shown that people with cataracts have a higher risk of dementia, and surgery could reverse this risk of dementia.^104^ Cataracts (62.6%) and refractive errors (19.7%) are among the most common causes of blindness in India.^105^ Given the very high prevalence of vision impairment in India and the potential to treat vision impairment effectively, there is a critical need for longitudinal research to clarify dementia risk among individuals with vision loss, as well as to assess whether restoring vision through surgery can reduce dementia risk in this population.

#### 3.2.13 Hearing loss

In the United States, the prevalence of hearing loss in individuals 35 years of age and older was reported to be 13.8%.^106^ Hearing loss affects 41% of the Indian population.^107^ The high cost of hearing aids and cochlear implants, combined with inadequate insurance coverage and limited government support, creates substantial barriers for those in need of essential auditory health care services. These challenges are particularly acute in rural India, where audiological services are sparse, and awareness about hearing health is limited.^108^

Hearing loss is independently associated with a higher risk of dementia.^8^ A meta‐analysis of prospective cohort studies from HICs shows that hearing loss is independently associated with a 59% higher risk of developing dementia, and doubles the risk of Alzheimer's disease.^109^ The Aging and Cognitive Health Evaluation in Elders (ACHIEVE) trial indicates that hearing aids may be effective in slowing cognitive decline among participants from the Atherosclerosis Risk in Communities (ARIC), who are at higher risk of cognitive decline, but not in the primary analysis.^110^ In the Indian context, the association between hearing loss and dementia is concerning. In a cross‐sectional study examining modifiable risk factors for dementia in India, the dementia score decreased with an increase in the percentage of hearing handicap.^111^ Despite the increasing burden, access to hearing care in India remains severely constrained. Yet, no longitudinal study has examined the relationship between hearing loss and dementia. This highlights a research gap and an important opportunity for future studies, especially given the modifiability of hearing impairment and the global evidence supporting its link with dementia.

#### 3.2.14 Traumatic brain injury (TBI)

Evidence from the HICs indicates that TBI significantly raises the risk of developing dementia and could result in its onset 2–3 years earlier compared to individuals without TBI.^112^, ^113^ The Global Burden of Disease Study 2019 reported that, between 1990 and 2019, India experienced an 11.1% rise in the age‐adjusted incidence of TBI and a 22.4% increase in its age‐adjusted prevalence.^114^ Among adults, road traffic accidents account for a major proportion of head injuries, followed by falls and violence in India, in contrast to HICs, where falls and violence are the major causes.^115^Although the burden of TBI is high and rising in India, we could not find studies that investigate the associations between TBI and dementia.^116^ This gap highlights the critical need for longitudinal studies to understand and mitigate the role of TBI in elevated dementia risk within the Indian population.

## 4 CONCLUSION

South Asians have higher PAF for dementia, and the PAFs for modifiable dementia risk factors, as compared to the HICs, support the hypothesis that targeting these risk factors could meaningfully contribute to dementia prevention at the population level.^31^ India, being the most populous nation in South Asia, presents a critical need for longitudinal research on dementia that accounts for its unique social and cultural contexts. Dementia prevalence and risk in India show significant differences and are reported to be lower compared to HICs. In contrast, cardiometabolic conditions such as hypertension, obesity, diabetes, low high‐density lipoprotein cholesterol (HDL‐C), and sleep disorders have a more pronounced effect on dementia risk for South Asians compared with Whites and other Asian groups. Although genetic variants identified through European ancestry appear to play a relatively limited role in dementia risk among South Asians, current evidence strongly suggests that modifiable risk factors have a far greater impact in this population. These findings offer a unique opportunity to investigate how these modifiable factors contribute to heightened dementia risk in Indians and to explore potential protective mechanisms underlying the lower dementia risk in the absence of these risk factors. They also suggest that interventions addressing lifestyle and cardiometabolic health may be particularly valuable for dementia prevention in this population.

Although factors such as alcohol and smoking have demonstrated strong associations with dementia risk in high‐income regions, Indian studies highlight the greater influences of low education, vision impairment, physical inactivity, social isolation, undernutrition, and untreated hypertension. Furthermore, understanding the gender‐specific factors within the Indian society is crucial for addressing the sex disparity in dementia prevalence. Although urban areas may have a greater burden of diagnosed disease, the higher rate of undetected conditions in rural settings highlights the critical need for improved screening and health care access to effectively address dementia risk in these populations. Other characteristic features in India, including the profound gender gaps in education, transitions from joint to nuclear families, high levels of indoor air pollution, and early‐life socioeconomic adversity, may shape the pathways linking these risks to cognitive decline differently than in Western cohorts. Long‐term studies are urgently needed to disentangle these factors and design culturally appropriate interventions that prioritize India's most relevant and modifiable risks.

The large Indian diaspora population worldwide plays a significant role in shaping how cultural adaptation influences health outcomes across various societies. Acculturation to new lifestyles, health care systems, and environmental exposures can alter dementia risk profiles, potentially modifying both protective and harmful factors compared with populations in countries of origin. Research and policy must address these global population movements and their effects by integrating biological, social, and environmental perspectives with culturally informed prevention and treatment strategies. Strengthening both India‐focused and diaspora research will help clarify how genetic, lifestyle, and metabolic factors influence dementia, guiding targeted prevention and care programs that reduce global disparities and ultimately advance brain health outcomes.

## 5 LIMITATION

A key limitation of this study is its focus on dementia as the primary outcome, rather than earlier stages of cognitive decline, such as MCI. MCI represents a critical transitional phase and is recognized increasingly as a more clinically actionable stage for implementing preventive and therapeutic interventions. By the time dementia is diagnosed, underlying neuropathological changes are often advanced, potentially limiting the effectiveness of interventions. However, dementia serves as a more robust and clinically well‐defined endpoint, particularly in population‐based and cross‐cultural studies, where variability in the diagnosis and classification of MCI remains a significant challenge. In resource‐limited settings like India, MCI is frequently underdiagnosed or inconsistently assessed, making dementia a more reliable and comparable outcome across populations.^117^ Thus, the use of dementia enhances the generalizability of our findings. Future research should incorporate earlier stages of cognitive impairment, including MCI, to better capture disease onset and inform timely, targeted prevention strategies.

## CONFLICT OF INTEREST STATEMENT

The authors declare that there are no conflicts of interest relevant to the research, authorship, and/or publication of this manuscript. Author disclosures are available in the Supporting Information.

## Supporting information



Supporting Information
